# Adverse pregnancy outcomes and long-term cardiovascular risk: lifelong connections

**DOI:** 10.1093/eurheartj/ehag654

**Published:** 2026-09-01

**Authors:** Veronica Giorgione, Nisha I Parikh, Daniela Pedicino, Basky Thilaganathan

**Affiliations:** Fetal Medicine Unit, St George's University Hospitals NHS Foundation Trust, London, UK; Women’s Heart Program, Department of Cardiology, Katz Institute for Women's Health, Long Island Jewish Medical Center, 270-05 76th Avenue, New Hyde Park, New York, NY 11040, USA; Department of Cardiovascular Medicine, Fondazione Policlinico Universitario A. Gemelli-IRCCS, Largo A. Gemelli 8, Rome 00168, Italy; Fetal Medicine Unit, St George's University Hospitals NHS Foundation Trust, London, UK; Vascular Biology Research Centre, Molecular and Clinical Sciences Research Institute, St George's University of London, London, UK

**Keywords:** Pregnancy, Adverse pregnancy outcomes, Cardiovascular disease, Fourth trimester, Postpartum, preeclampsia, gestational diabetes

## Abstract

Pregnancy represents a critical period during which the maternal cardiovascular system adapts to profound haemodynamic and metabolic demands. When these adaptive mechanisms are impaired, adverse pregnancy outcomes (APOs)—including hypertensive disorders of pregnancy, gestational diabetes, pre-term birth, small-for-gestational-age birth, and pregnancy loss—occur. Extensive epidemiological evidence demonstrates that APOs are not isolated obstetric events but early clinical markers of cardiometabolic dysfunction that identify women at increased risk of coronary artery disease, heart failure, stroke, chronic kidney disease, and premature cardiovascular mortality. Shared mechanisms include endothelial dysfunction, inflammation, and insulin resistance, establishing a continuum between obstetric complications and later cardiovascular disease. Early post-partum studies reveal persistent hypertension, adverse cardiac remodelling, and metabolic abnormalities that mediate much of the long-term excess cardiovascular risk. These findings redefine the ‘fourth trimester’ as a preventive window in which timely blood pressure surveillance, metabolic screening, and lifestyle optimization can yield durable cardiovascular benefit. Integrated cardio-obstetric care models, digital follow-up, and systematic transition to primary care are crucial for maintaining long-term prevention. Addressing persistent gaps in limited provider awareness, fragmented follow-up, and disparities across socially disadvantaged populations remains critical. Recognizing pregnancy complications as indicators of underlying cardiovascular vulnerability offers an opportunity to shift prevention upstream and promote lifelong cardiovascular health for women.

## Graphical Abstract

**Graphical Abstract:**
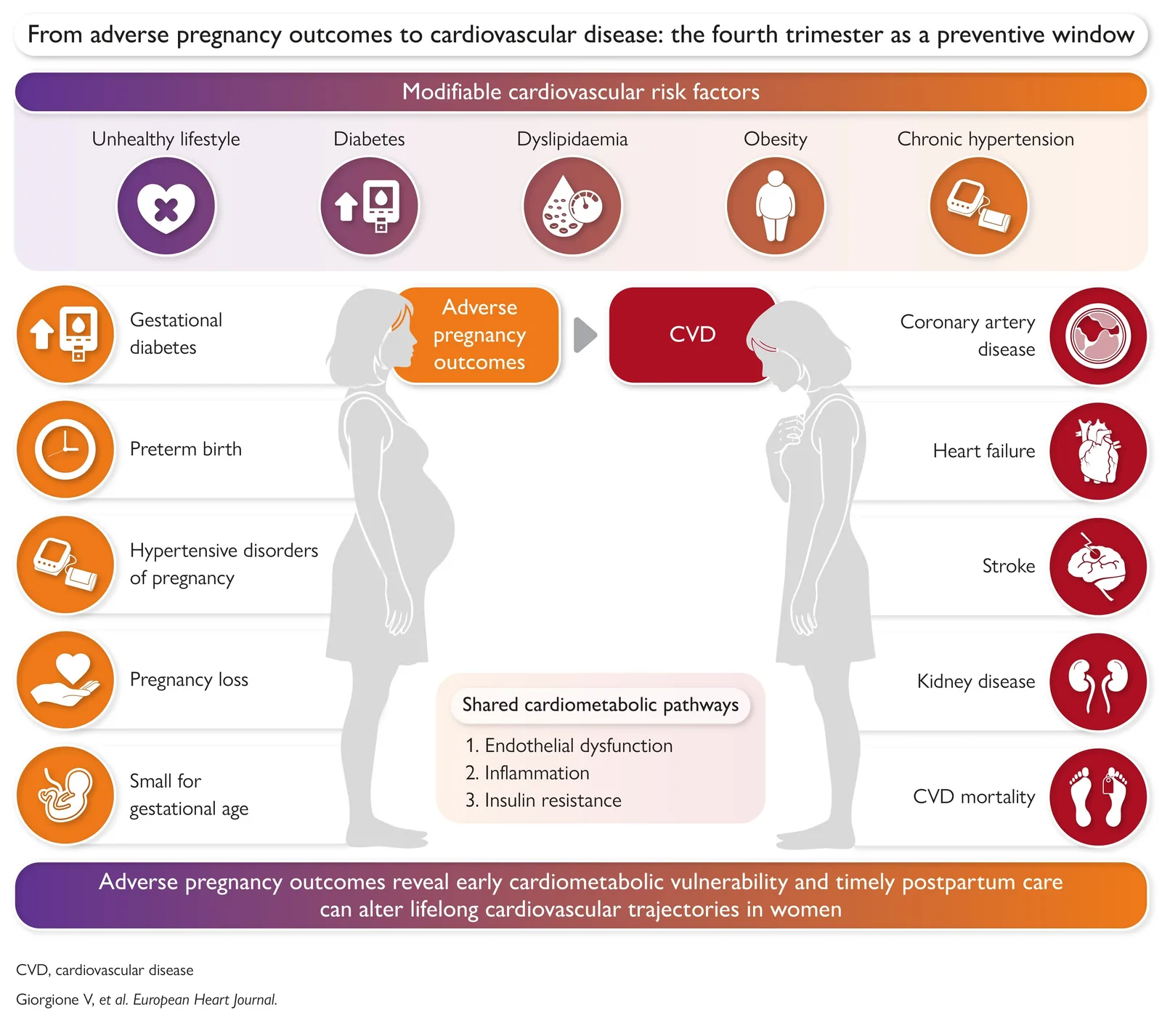
From adverse pregnancy outcomes to cardiovascular disease: the fourth trimester as a preventive window. Conceptual graphical abstract linking adverse pregnancy outcomes (left) to later cardiovascular disease (CVD) (right) through the emergence of modifiable cardiovascular risk factors (centre), such as chronic hypertension, obesity, dyslipidaemia, diabetes and unhealthy lifestyle behaviours. Downstream cardiovascular manifestations include coronary artery disease, heart failure, stroke, kidney disease, and CVD-related mortality. Recognition of this continuum highlights the fourth trimester as a key opportunity for prevention and long-term cardiovascular health promotion in women. Potential postnatal interventions include home blood pressure monitoring during the first 3 postpartum months, review in a dedicated cardio-obstetric clinic at 3–6 months, and transition to primary care with annual cardiometabolic assessment thereafter. CVD, cardiovascular disease, HBPM home blood pressure monitoring.

## Introduction

Pregnancy offers a unique temporal view of how the cardiovascular system responds to substantial haemodynamic and metabolic stress. When maternal physiological adaptation to haemodynamic and metabolic pregnancy-induced stress is impaired, the risk of adverse pregnancy outcomes (APOs) such as hypertensive disorders of pregnancy (HDP), gestational diabetes (GDM), preterm birth, small for gestational age (SGA) birth and pregnancy loss is significantly increased. A substantial body of epidemiologic literature has linked APOs to persistent cardiometabolic vulnerability after birth, with excess risks of coronary artery disease (CAD), heart failure (HF), stroke, chronic kidney disease (CKD), and cardiovascular mortality in these mothers. Overall, APOs should be considered early markers of cardiometabolic vulnerability and incorporated into cardiovascular risk assessment and long-term prevention strategies in women.

The early postpartum period after APOs makes this trajectory visible: chronic hypertension and adverse left ventricular remodelling with diastolic impairment do not fully resolve in a significant proportion of women.^1–3^ These observations recast the ‘fourth trimester’, which refers to the first 12 weeks after childbirth, as an actionable preventive window. Once pregnancy-related haemodynamic stress has resolved, persistent hypertension and subclinical cardiac dysfunction can be more clearly identified, distinguishing transient gestational changes from underlying cardiovascular vulnerability. This period is particularly important because women are still engaged with healthcare services, offering a unique opportunity for structured blood-pressure (BP) surveillance, cardiovascular risk assessment, and early intervention in a population of adults at significantly increased risk of cardiovascular disease (CVD)—the leading cause of death in women.^4^ Given this context, a history of APOs may be considered an early warning signal prompting systematic screening for modifiable risk factors and timely intervention to address them and prevent CVD.

This review summarizes current evidence with four aims: (i) to quantify the magnitude of cardiovascular risk after specific APOs; (ii) to outline shared pathophysiology linking APOs and CVD; (iii) to chart the temporal course of the cardiovascular legacy from early postpartum cardiovascular findings to later organ damage; and (iv) to translate these insights into pragmatic strategies for prevention, covering optimal fourth-trimester care and the system gaps that impede its delivery.

## Cardiovascular risk after adverse pregnancy outcomes

### Hypertensive disorders of pregnancy

HDP, particularly preeclampsia (PE), confer a marked and clinically meaningful excess risk of later CAD, HF, and stroke (*Table 1*).^2,5–9^ The magnitude of association is comparable to that of traditional CVD risk factors such as smoking or diabetes (*Figure 1*).^14,35–38^ HF risk emerges early-within the first years postpartum-and remains elevated for decades, with a particular link to HF with preserved ejection fraction (HFpEF).^10–14,39^ Stroke risk is increased for both ischaemic and haemorrhagic subtypes and may persist long term.^2,5,6,15–19^ Cardiovascular maternal mortality is also higher after HDP, especially in women with early-onset or recurrent disease.^11,21,22^

**Figure 1:**
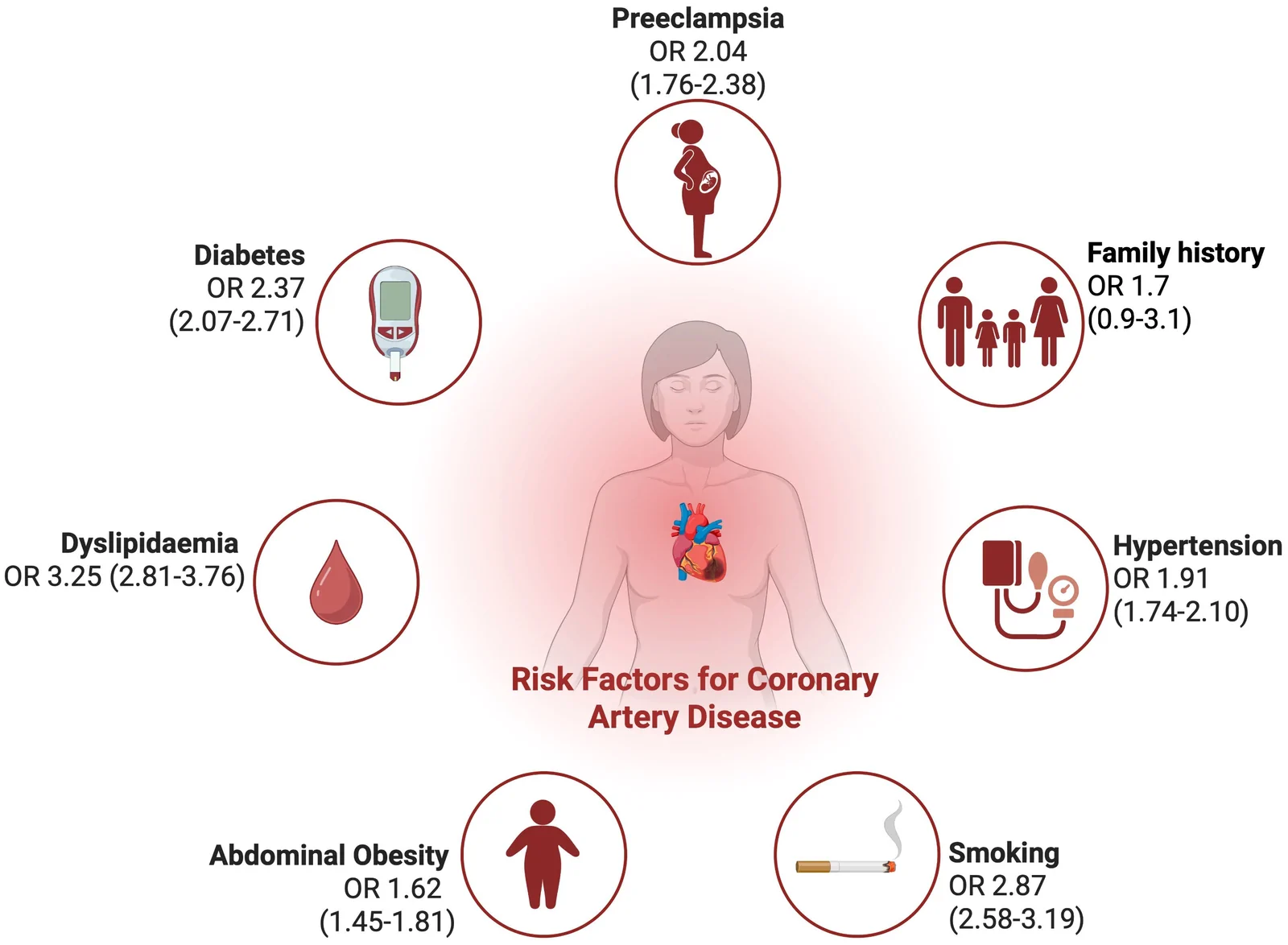
Cardiovascular risk factors: adverse pregnancy outcomes vs traditional risk factors. Odds ratio (OR) with 95% confidence intervals (CI) estimates for smoking, diabetes, hypertension, dyslipidaemia, abdominal obesity, psychosocial stress, diet, and physical inactivity are taken from the INTERHEART study.^38^ OR estimates for preeclampsia are from a recent meta-analysis by Inversetti *et al*.^6^ Estimates for family history of premature cardiac heart disease are from Lloyd-Jones *et al*.^37^ Estimates are presented for illustrative comparison only and originate from different studies with varying populations and adjustment strategies; therefore, they should not be interpreted as directly comparable measures of independent cardiovascular risk. Created in https://BioRender.com

**Table 1:** Maternal cardiovascular risk after adverse pregnancy outcomes

Adverse pregnancy outcomes	Coronary artery disease	Heart failure	Stroke	Cardiovascular mortality	Key notes
HDP	∼2-fold ↑^2,5,6^	∼2-fold ↑ from first years postpartum^6,10–13^ Strong association with HFpEF^14^	∼2-fold ↑ from first years postpartum^2,5,6,15–19^ Increased risk of both ischaemic and haemorrhagic stroke^16^	1.7–2.0-fold ↑^6,11,20^	Dose–response with recurrent/early-onset PE^7–9,12,13,21,22^
GDM	∼2-fold ↑^23^	∼1.6-fold ↑^24^	∼1.4–1.5-fold ↑^24^	> 2-fold ↑^11^	
Spontaneous Preterm birth	∼1.4-fold ↑^25^		∼1.7-fold ↑^17,25^	∼1.3-fold ↑ risk^26^	Dose–response with gestational age at delivery^17,27,28^
SGA	Modest association^29^			∼1.7-fold ↑^11^	Amplified risk with concurrent HDP
Pregnancy loss (miscarriage and stillbirth)	∼1.3–1.5-fold ↑^30,31^		∼1.1–1.4-fold ↑^31,32^	∼2-fold ↑ if ≥3 losses	Dose–response with recurrence; stronger association after stillbirth^30,32–34^

HDP, hypertensive disorders of pregnancy; PE, preeclampsia; GDM, gestational diabetes; T2D, type 2 diabetes; SGA, small for gestational age; CAD, coronary artery disease; HF, heart failure; HFpEF, HF with preserved ejection fraction; CVD, cardiovascular disease.

Effect sizes are presented as approximate relative risk increases to facilitate comparison across outcomes; original estimates were reported as hazard ratios, relative risks, or odds ratios in the cited studies. Estimates derive from heterogeneous studies with different adjustment models and should be interpreted as overall associations rather than directly comparable measures of risk independent of conventional cardiovascular risk factors.

### Gestational diabetes

Women with prior GDM are consistently reported to have higher risks of overall CVD, including CAD, HF, and cerebrovascular disease (*Table 1*).^20,23,24,40^ Although part of this excess risk is mediated by subsequent type 2 diabetes (T2D), associations persist even after adjustment for later diabetes, suggesting that GDM identifies a broader cardiometabolic vulnerability. National registry data further demonstrate an increased risk of cardiovascular maternal mortality among women with prior GDM.^11^

In addition, delivery of a large-for-gestational-age infant may identify women with underlying metabolic dysfunction, potentially reflecting unrecognized dysglycaemia or undiagnosed GDM, and has been associated with a less favourable maternal cardiometabolic risk profile in some studies; however, evidence remains limited and inconsistent.^41^

### Preterm birth

Both iatrogenic and spontaneous preterm birth are associated with increased long-term maternal CVD risk (*Table 1*).^27,42^ Spontaneous preterm birth, which more commonly reflects underlying inflammatory, thrombotic, or vascular pathology, may identify a distinct maternal phenotype with greater intrinsic cardiovascular susceptibility compared with iatrogenic preterm birth, which reflects fetal or placental rather than primarily maternal disease.^27,28^ Meta-analyses and large population-based cohorts show higher rates of ischaemic heart disease and stroke after preterm delivery, with a clear dose–response relationship according to gestational age at birth.^17,25,28^ Excess cardiovascular maternal mortality has also been reported.^11,26,43^

### Small-for-gestational-age birth

Delivery of an SGA infant has been linked to later maternal CVD, particularly atherosclerotic disease and stroke (*Table 1*).^29,44^ Although earlier meta-analyses reported modest and heterogeneous associations,^20^ more recent large-scale cohort studies demonstrate increased long-term cardiovascular maternal mortality, especially when SGA coexists with HDP.^11,45,46^ Recurrent delivery of very low birth weight infants has been associated with a greater burden of maternal cardiovascular risk.^47^

### Pregnancy loss

Miscarriage and stillbirth have also been associated with increased maternal CVD risk (*Table 1*).^20,30,32^ The excess appears greater with recurrent pregnancy loss, with several studies reporting substantially higher risks of myocardial infarction, stroke, and cardiovascular maternal mortality among women with multiple losses.^30,32–34^ Stillbirth, in particular, is associated with a clear elevation in subsequent cardiovascular events and maternal mortality.^30–32^

### Multiple APOs

While each APO is independently associated with an increased risk of future CVD, women experiencing multiple distinct APOs appear to represent a particularly high-risk subgroup, supporting the concept of a cumulative burden of adverse pregnancy experiences and underlying cardiometabolic vulnerability.^48,49^

## Pathophysiology of the postpartum cardiovascular legacy

### Common pathophysiological pathways linking APOs and CVD

APOs are heterogeneous conditions that arise through distinct, although often overlapping, pathophysiological mechanisms. While GDM is primarily characterized by metabolic dysregulation and insulin resistance, many other APOs, particularly placenta-mediated complications such as HDP and foetal growth restriction, are associated with uteroplacental hypoperfusion, resulting in placental hypoxia and oxidative stress.^50^ In turn, these changes can induce an imbalance in vasoactive substances with excess of soluble fms-like tyrosine kinase 1 (sFlt-1) and reduced placental growth factor (PlGF), initiating the molecular cascade resulting in the signs of PE. The downstream consequence is endothelial dysfunction, clinically manifest in multiple organs through hypertension, renal impairment, hepatic involvement and neurological symptoms, often accompanied by coagulopathy due to endothelial activation and platelet aggregation.^51^ Endothelial dysfunction is shared as a central mechanism in CVD, where impaired nitric oxide signalling, vascular stiffening, and reduced microvascular density promote atherosclerosis and HFpEF.^52^ Accompanying systemic low-grade inflammation amplifies risk, as extensively described in HFpEF, drives myocardial stiffening and coronary microvascular dysfunction, and contributes to the female predominance of this condition.^53^ Finally, insulin resistance is frequently observed in women with APOs and is known to aggravate oxidative stress, inflammation, and endothelial injury.^54–56^ Beyond pregnancy, insulin resistance is recognized as a major determinant of CVD, fuelling dyslipidaemia, hypertension, and metabolic cardiac dysfunction.^56^ Data from the STEP-HFpEF trial further illustrate the metabolic dimension of HFpEF: treatment with the GLP-1 receptor agonist semaglutide produced significant improvements in symptoms and exercise capacity in patients with obesity-related HFpEF—a phenotype that overlaps mechanistically with the cardiac dysfunction observed after APOs, though direct evidence in postpartum populations is lacking.^57^ Emerging genetic evidence further supports the concept that APOs and later CVD may share common biological susceptibility pathways, including genetic determinants of blood pressure regulation, endothelial dysfunction, and cardiometabolic health.^58–60^ However, conflicting signals in some studies highlight an unresolved mechanistic question that future studies using multi-ancestry genomic data will need to address.^59^

### Cardiovascular phenotype in women with APOs

Women who develop APOs already show subtle cardiovascular alterations before conception, including higher baseline BP, lower cardiac output, and higher systemic vascular resistance.^61–63^ Higher blood pressure trajectories from early pregnancy have been associated with a significantly higher risk of developing HDP.^64^ Haemodynamic studies have also shown that some women exhibit elevated total peripheral vascular resistance despite having a BP within the normal range, and this high-resistance phenotype is strongly linked to the later development of HDP and SGA birth.^65^ In women known to be at high risk of HDP, maternal transthoracic echocardiography in early pregnancy reveals concentric remodelling and evidence of diastolic impairment, indicating maladaptive cardiac changes well before clinical HDP develops.^66,67^ In women presenting with HDP, echocardiographic studies consistently demonstrate increased left ventricular mass, concentric remodelling, impaired diastolic function, and reduced global longitudinal strain, indicating maladaptive cardiac mechanics beyond that seen with hypertension outside pregnancy.^68,69^ Circulating cardiovascular biomarkers are also elevated in HDP, with higher NT-proBNP concentration compared with normotensive pregnancies, and these findings overlap with biomarker profiles described in HFpEF.^70,71^

### Cardiovascular risk factors before and after pregnancies complicated by APOs

APOs and CVD share a common upstream set of risk factors, reflecting a unifying cardiometabolic predisposition.^72,73^ A meta-analysis of pre-pregnancy maternal health demonstrated markedly increased risks of HDP in women with renal disease (RR 7.76), chronic hypertension (RR 3.68), type 2 diabetes (RR 3.57), and obesity (RR 2.65).^74^ The prevalence of APOs has risen over recent decades in parallel with the increasing burden of obesity and hypertension in women of reproductive age.^75,76^ In the CARDIA cohort, less optimal pre-pregnancy cardiovascular health assessed by the American Heart Association’s Life’s Essential 8 score, which integrates four health behaviours (diet, physical activity, nicotine exposure, and sleep) and four clinical factors (body mass index [BMI], BP, blood lipids, and blood glucose), was strongly associated with the risk of subsequently developing HDP.^77^ Women with moderate (50–79 points) and low (<50 points) cardiovascular health categories had 1.8- and 4-fold higher odds, respectively, of developing HDP compared with those with high cardiovascular health (≥80 points).^77^ Polycystic ovary syndrome, a common pre-pregnancy cardiometabolic disorder characterized by insulin resistance and androgen excess, clusters with obesity, dysglycaemia and hypertension and is associated with a higher risk of HDP and with increased long-term cardiovascular morbidity.^78,79^ A similar clustering of pre-pregnancy risk factors has been reported for other APOs.^80,81^ (*Figure 2*)

**Figure 2:**
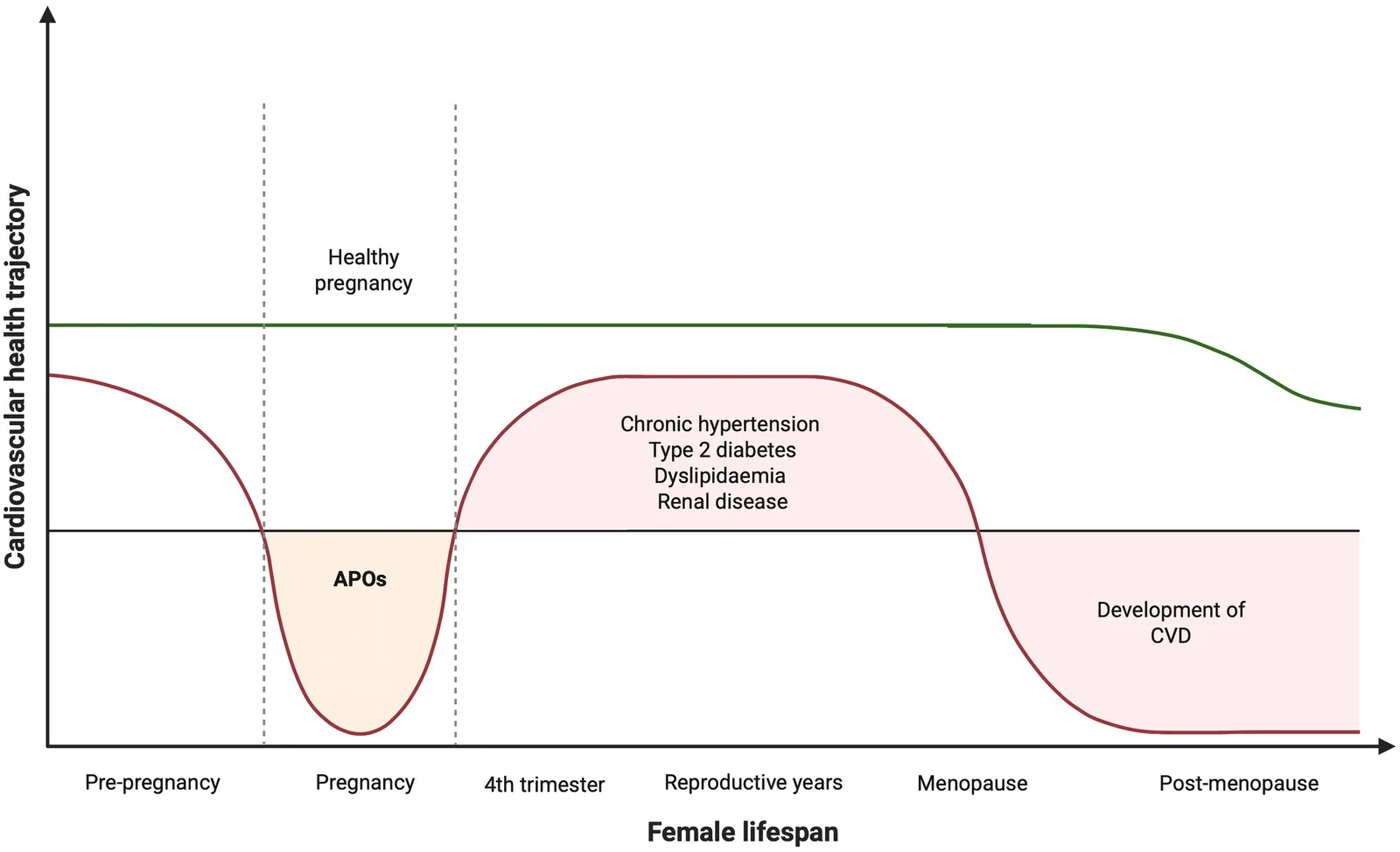
Maternal cardiovascular health before, during and after APOs. The green line depicts a healthy pregnancy as a transient physiological stress with preservation of global cardiovascular health. The red line illustrates pregnancies complicated by adverse pregnancy outcomes (APOs), showing an acute decline during gestation with only partial recovery in the 4th trimester, followed by accelerated accumulation of risk factors (chronic hypertension, type 2 diabetes, dyslipidaemia, renal disease) and earlier transition to overt cardiovascular disease (CVD) in later life. Created in https://BioRender.com

After delivery, the high-risk cardiovascular phenotype persists in women with a history of APOs. In the HUNT study, which followed more than 23 000 women across three decades, those with a history of HDP had systolic BP trajectories that were on average 5–10 mmHg higher and diastolic pressures 3–5 mmHg higher from early adulthood through midlife compared with women with normotensive pregnancies.^82^ Furthermore, additional analyses from the HUNT cohort demonstrated that both pregnancy loss and delivery of a SGA infant were associated with a higher subsequent risk of developing chronic hypertension and other cardiovascular risk factors, reinforcing the concept that a broad range of APOs may reflect an underlying predisposition to CVD.^41,83^ The association between adverse pregnancy outcomes and postpartum cardiovascular risk factors may also explain why adding APOs to conventional CVD risk calculators yields only modest improvements in prediction.^84,85^ In the MuM-PreDiCT study, although pregnancy-related complications were independently associated with future CVD, their inclusion provided only limited incremental predictive value beyond established cardiometabolic risk factors, likely because much of the excess risk is already reflected in those cardiovascular risk factors.^84^

Longitudinal data indicate that the excess CVD risk after APOs is mediated largely by the development or persistence of conventional cardiovascular risk factors over time. In a prospective analysis of more than 60 000 women from the Nurses’ Health Study II, mediation analyses demonstrated that established CVD risk factors accounted for 57% of the excess CVD risk associated with PE and 84% of that associated with gestational hypertension.^86^ In line with this, emerging evidence shows that the association between APOs and later CVD reflects a pre-existing cardiovascular vulnerability and is only partly attributable to the pregnancy complication itself. In the HAPO Follow-Up Study including 4269 women reassessed a mean of 11.6 years after delivery, pre-pregnancy obesity was strongly associated with higher midlife BP (+7 mmHg), triglycerides (+28.5 mg/dL), and HbA1c (+0.3%), with mediation analyses showing that GDM explained ∼25% of the excess glycaemic risk and new HDP ∼12% of the excess BP risk.^87^ In a prospective cohort of 1143 women from the CARDIA study, those with poor pre-pregnancy cardiovascular health had a significantly higher risk of developing GDM and, two decades later, a greater prevalence of coronary artery calcium. Mediation analyses showed that GDM accounted for only ∼6% of the association between poor cardiovascular health and later coronary artery calcium, highlighting that most of the excess risk was explained by suboptimal cardiovascular health pre-pregnancy.^80^ Similarly, a large UK electronic health record cohort showed that women with a history of APOs exhibited distinct adverse cardiovascular risk profiles both before and after pregnancy.^88^ Their findings indicate that adverse trajectories of blood pressure and other cardiometabolic risk factors often predate the index pregnancy and persist or widen across the life course, suggesting an underlying cardiovascular vulnerability. These findings support the interpretation that APOs function primarily as early clinical markers of an underlying cardiometabolic predisposition, rather than as isolated causal events. This view is not universally accepted, as an alternative position holds that the haemodynamic and inflammatory insults of APOs themselves accelerate cardiovascular injury through mechanisms beyond those captured by conventional risk factors.^48^ It is this latter hypothesis that motivates postpartum intervention trials regardless of whether the APO is causal or reflective. Regardless of this ongoing debate, the practical implication of both positions converges to justify the structured cardiovascular follow-up of women with APOs. The causal vs marker distinction shapes how intensively clinicians should counsel women about pregnancy-attributable risk, how intervention trials should be designed, and whether preventing the APO through preconception optimization should be prioritized alongside postpartum care.

## Temporal course of the postpartum cardiovascular legacy

### Early postpartum cardiovascular risk profile (fourth trimester and early years)

In the months following delivery, women with APOs frequently show persistence or early manifestation of CVD risk factors such as hypertension, dyslipidaemia and impaired glucose metabolism.^89^ These early abnormalities create the substrate that explains the subsequent development of clinical CVD in later life. Women with HDP have a 5 to 8-fold higher risk of developing chronic hypertension, with incident hypertension detectable within just weeks to months postpartum.^90^ Consistent findings come from large population-based cohorts such as a Danish registry of 1.5 million primiparous women; those with HDP had a 4 to 10-fold higher adjusted risk of chronic hypertension within five years postpartum. A French national study on 2.6 million women with a mean postpartum follow-up of three years confirmed that the risk of hypertension remained elevated after adjustment for pre-existing cardiovascular risk factors for all types of HDP.^91,92^ Furthermore, up to nearly two-thirds of women with a history of HDP may have persistent chronic hypertension within the first year postpartum, highlighting the importance of very early postpartum cardiovascular assessment.^3,55,93^

Metabolic syndrome, defined by the coexistence of obesity, hypertension, dysglycaemia, and dyslipidaemia, occurs in ∼20% of women with prior HDP within 1–3 years postpartum, corresponding to a three-fold higher risk compared with controls. The risk is greatest after early-onset HDP, where prevalence exceeds 25%—nearly five-fold higher than in age-matched women from the general population.^54,94^ Renal impairment, which is a common clinical manifestation of HDP, and its consequences may persist beyond pregnancy. In a prospective observational multicentre study including 237 women with HDP and acute kidney injury (AKI), up to one-third failed to recover baseline renal function by discharge, with recovery rates decreasing by AKI stage.^95^ Population-based data further show that the risk of CKD is highest in the first five years and is substantially higher after preterm PE (RR 3.93, 95% CI 2.90–5.33) compared with term disease (RR 2.27, 95% CI 2.02–2.55).^96^ Subclinical renal abnormalities detected by magnetic resonance imaging performed 6–12 months postpartum, including smaller kidney volumes after HDP, were related to lower renal function at delivery, and reduced corticomedullary differentiation, which may represent an underlying mechanism for the increased risk of renal damage later in life.^97^

### From APOs to long-term organ damage: cardiac, vascular, and multi-organ remodelling

While epidemiological studies consistently demonstrate an increased risk of HF, CAD, stroke and CKD after APOs, accumulating mechanistic and imaging evidence suggests that these events are preceded by persistent subclinical organ damage affecting the heart, vasculature, brain and kidneys.

Women with prior HDP show a higher prevalence of left-ventricular hypertrophy in midlife, consistent with persistent adverse remodelling, diastolic dysfunction, impaired left- and right-ventricular strain, and subclinical HF (stage B) at a relatively young age.^98–101^ At 8–10 years postpartum, coronary microvascular dysfunction is more frequent after HDP, indicating impaired myocardial perfusion despite unobstructed epicardial arteries.^102^ A recent meta-analysis further demonstrated that a history of HDP was associated with a significantly higher prevalence of coronary artery calcium, with an OR of 1.57 (95% CI 1.39–1.77).^103^ These findings are supported by systematic evidence showing that abnormal cardiac geometry, diastolic dysfunction, and impaired strain can persist for years after delivery. Collectively, these data highlight a continuum from persistent remodelling and microvascular dysfunction from HDP through to overt HF and CAD in later life.^101^

HDP also has lasting detrimental effects on the brain, with women exhibiting increased risks of stroke, cognitive decline, and dementia at a relatively young age.^44,104,105^ The blood–brain barrier dysfunction is increasingly recognized as a hallmark of HDP and may serve as an early indicator of long-term cerebrovascular vulnerability.^106^ Ultra-high-field cerebral 7 Tesla magnetic resonance imaging demonstrated global blood–brain barrier impairment, with persistently elevated leakage rates in both grey and white matter detectable up to 5–8 years postpartum.^107^ Furthermore, white matter lesions are detectable during pregnancy in women with HDP and remain approximately two-fold more prevalent for at least 7 years postpartum.^108,109^ These lesions co-occur with alterations in white matter microstructure, particularly in the temporal lobes, and with reduced grey matter integrity and total brain volume.^110–112^ This evidence reinforces the view that cerebrovascular injury due mainly to cerebral small vessel disease extends well beyond the peripartum period.

APOs are also associated with long-term renal damage, in particular end-stage renal disease. Women with PE had the highest risk (HR 4.7, 95% CI 3.6–6.0), which rose dramatically with recurrent disease (RR 15.5, 95% CI 7.8–30.8).^113^ Persistent microvascular damage, nephrosclerosis, and fibrosis may create a substrate for CKD and progression to end-stage renal disease. This trajectory is amplified by later-life hypertension and metabolic stress, which are more common after HDP.^114^

## Prevention strategies and clinical management

### Optimal postpartum care: seizing the fourth trimester

The ‘fourth trimester’ is a critical window not only because of the acute risk of medical complications but also because it offers an opportunity to shape long-term maternal cardiovascular health. BP often peaks within the first week after delivery, and unchecked fluctuations can precipitate severe hypertension, stroke, or HF, underscoring the importance of early postpartum BP monitoring.^115^ Most obstetricians continue to treat persistent hypertension within the first 4 weeks postpartum, initiating or adjusting therapy at thresholds of systolic BP ≥150 mmHg or diastolic BP ≥100 mmHg.^116,117^ However, in the absence of robust evidence, it may be reasonable to adopt postpartum BP targets consistent with those for non-pregnant, age-matched adults, given that the foetus is no longer at risk of placental hypoperfusion, while ensuring that antihypertensive therapy is compatible with lactation safety.^118^ This suggestion is underscored by the fact that elevated BP definitions and thresholds for treatment have been lowered with each iteration of recent hypertension guidelines. The American College of Obstetricians and Gynecologists (ACOG) recommends BP checks within 72 h and again at 7–10 days postpartum, whereas National Institute for Health and Care Excellence (NICE) advises a more structured schedule with daily measurements for the first 2 days, at least once daily for days 3–5, a 2-week review if on antihypertensives, and a comprehensive assessment at 6–8 weeks.^116,117^

The POP-HT randomized trial provides clear evidence that home BP monitoring (HBPM) and self-management via physician-guided antihypertensive titration in the fourth trimester after HDP yield both short- and longer-term cardiovascular benefits (*Figure 3*). This approach achieved significantly lower 24-hour ambulatory BP at 9 months compared with usual care (−6.5/−5.8 mmHg for systolic BP/diastolic BP), despite a median antihypertensive use of only 39 days following delivery.^119^ The cardiac imaging sub-study of this trial demonstrated favourable remodelling, with reduced left ventricular mass, smaller chamber volumes, and improved systolic and diastolic function in the intervention group compared with usual care.^120^ The study also showed improvements in central arterial stiffness with a significant reduction in aortic diastolic BP (−5.2 mm Hg, 95% CI, −8.0 to −2.2) and pulse wave velocity (−0.71 m/s, 95% CI −1.42 to −0.006).^121^ These findings establish that BP self-monitoring combined with physician-guided medication titration for up to 3 months in the immediate postpartum period not only might mitigate acute risk but also produce durable improvements in cardiovascular structure and function, with implications for the long-term prevention of CVD in this high-risk population.

**Figure 3:**
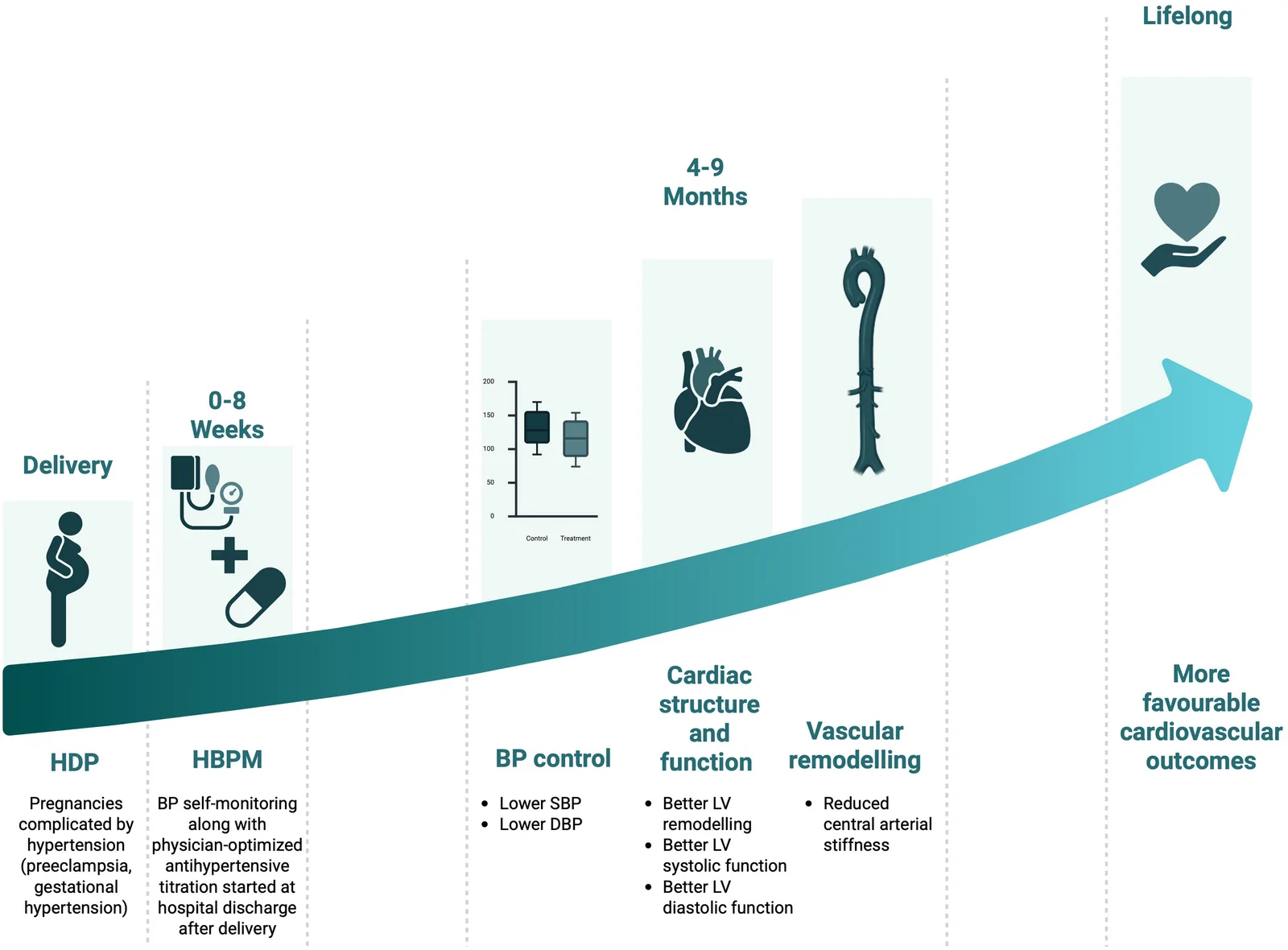
Home blood pressure monitoring: bridging postpartum care to lifelong prevention. Conceptual representation of the potential trajectory from delivery to improved lifelong cardiovascular outcomes through structured postpartum hypertension management. In pregnancies complicated by hypertensive disorders of pregnancy (HDP)—including preeclampsia and gestational hypertension—implementation of home blood pressure monitoring (HBPM) with physician-guided antihypertensive titration during the first 0–8 weeks postpartum leads to improved blood pressure (BP) control, with lower systolic (SBP) and diastolic (DBP) values. Over subsequent months (4–9 months postpartum), better left ventricular (LV) remodelling, enhanced systolic and diastolic function, and reduced central arterial stiffness are observed, reflecting favourable cardiac and vascular remodelling.^119–121^ These improvements are expected to translate into more favourable long-term cardiovascular outcomes. Created in https://BioRender.com

Cardiometabolic screening and lifestyle modifications are equally essential in the postpartum period for women with APOs. Scientific statements recommend that high-risk women undergo comprehensive cardiovascular health (CVH) screening, including BP, lipids, glucose, and weight/obesity assessment.^48,122^ However, the optimal timing and mode of testing remain uncertain, as physiological changes of pregnancy persist beyond delivery: cholesterol, glucose, and BP values may not return to baseline until at least 12 weeks postpartum (*Figure 4*). More specific recommendations include blood glucose monitoring for women with a history of GDM. The American Diabetes Association advises a 2-hour 75-g oral glucose tolerance test at 4–12 weeks postpartum, yet uptake remains low.^123,124^ In the UK, routine practice in line with the NICE guideline is to perform an HbA1c test at 12 weeks postpartum in women with prior GDM, followed by annual HbA1c screening thereafter.^125^ Obtaining a baseline lipid panel within the first postpartum year is reasonable to identify familial hypercholesterolaemia and stratify CVD after APOs.^122^ Lipid levels typically normalize by around 3 months postpartum and are minimally affected by lactation or oral contraceptives.^126^ Lifestyle counselling should be a cornerstone of postpartum prevention after APOs. Evidence from multiple postpartum randomized controlled trials based on the original Diabetes Prevention Program shows that structured interventions, such as behavioural strategies like goal setting, self-monitoring, dietary changes in fat and calorie intake, and engaging in at least 150 min of moderate-intensity physical activity per week, can achieve clinically meaningful weight loss at 6–12 months postpartum and improve metabolic risk.^122,127–129^ Among women with recent HDP, digital interventions using gamification increased physical activity by an average of 647 steps/day compared with usual care and were associated with enhanced awareness of cardiovascular risk, encouraged healthier eating, and reduced sedentary behaviour.^130,131^

**Figure 4:**
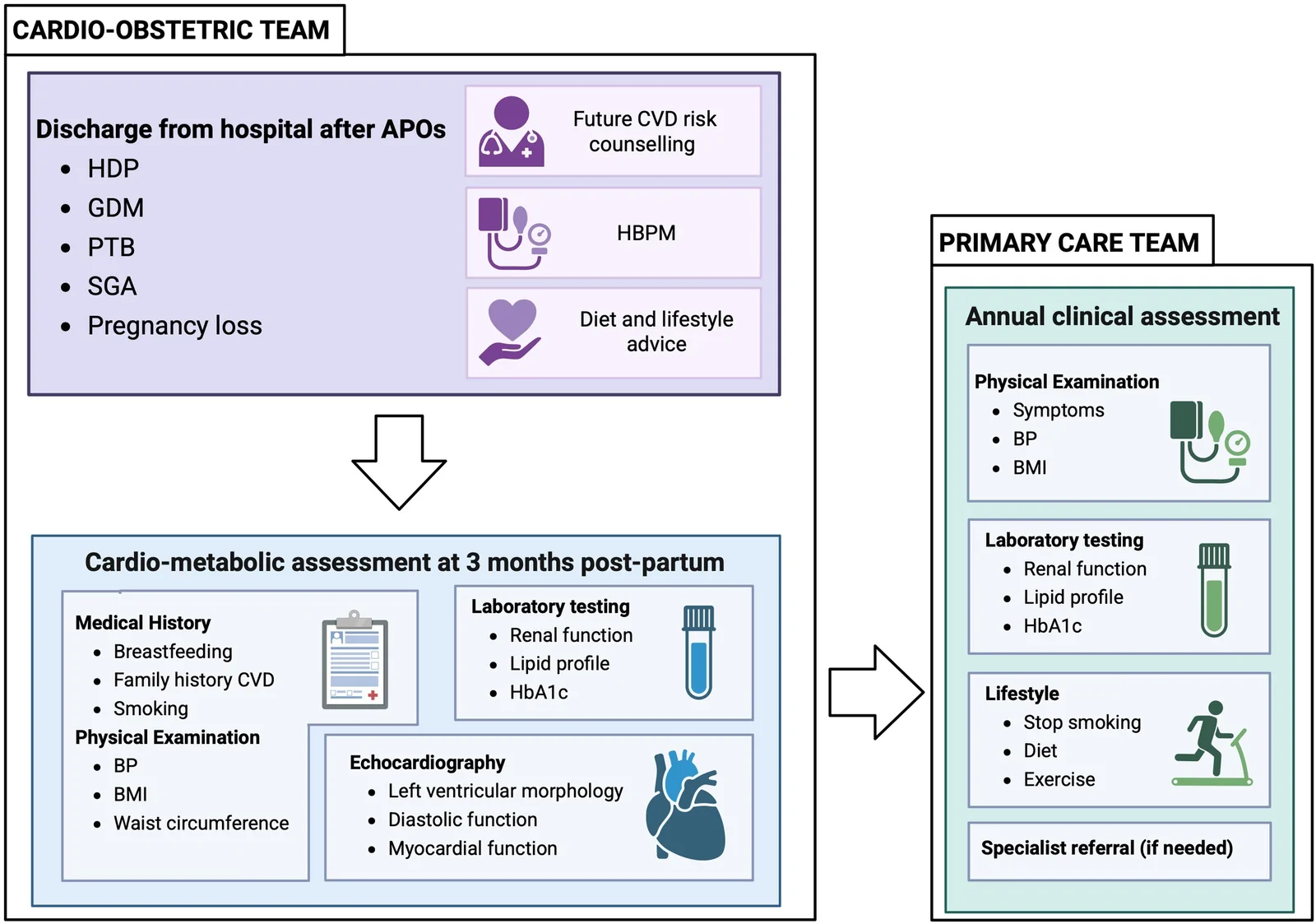
Optimal care pathway after APOs since the fourth trimester. Schematic representation of a structured care pathway linking the cardio-obstetric and primary care teams to ensure early cardiovascular risk assessment and long-term surveillance in women with prior adverse pregnancy outcomes (APOs). At hospital discharge after hypertensive disorders of pregnancy (HDP), gestational diabetes mellitus (GDM), preterm birth (PTB), small-for-gestational-age (SGA) infants, or pregnancy loss, patients should receive counselling on future cardiovascular disease (CVD) risk, home blood pressure monitoring (HBPM), and diet/lifestyle advice. A comprehensive cardio-metabolic evaluation is recommended at three months postpartum, including medical history (breastfeeding, family history of CVD, smoking), physical examination (blood pressure [BP], body mass index [BMI], waist circumference), laboratory testing (renal function, lipid profile, glycated haemoglobin [HbA1c]), and echocardiography to assess left ventricular morphology and diastolic and myocardial function. Thereafter, annual clinical review in primary care should include physical examination, laboratory tests, and lifestyle optimization, with specialist referral as indicated. Created in https://BioRender.com

### Preconception cardiovascular optimization

Recent AHA scientific statements and population-based cohort data propose that the most impactful intervention window may precede pregnancy entirely.^81^ This preconception-first perspective holds that women who enter pregnancy with optimized cardiovascular health have substantially lower rates of APOs and a more favourable long-term cardiometabolic trajectory, regardless of pregnancy outcome.^77,80^ Under this framework, postpartum care is necessary but inherently reactive, as it addresses risk that has already materialized as a pathological pregnancy outcome. The preconception period, by contrast, offers the opportunity to reduce APO incidence itself, thereby shrinking the population that requires postpartum cardiovascular surveillance.^81^ These two positions are not mutually exclusive as preconception optimization and fourth-trimester intervention address different points on the same risk continuum with different resource and policy implications. Investment concentrated in postpartum care leaves the underlying burden of cardiometabolic vulnerability in reproductive-age women largely unaddressed. A coherent prevention strategy requires both upstream preconception engagement and structured postpartum follow-up, with primary care and women's health services taking joint responsibility across the reproductive life course.^132,133^

### Integrated care pathways between obstetrics and cardiology

The optimal model of postpartum care is a dedicated cardio-obstetric clinic, where obstetricians, cardiologists, and allied health professionals jointly evaluate women with APOs. These clinics should provide timely BP monitoring and antihypertensive titration, comprehensive cardiovascular risk assessment (lipids, glucose, renal function), and structured lifestyle counselling, while also addressing reproductive health and mental well-being.^115^ Multidisciplinary care ensures that the cardiovascular implications of APOs are systematically recognized and managed from the earliest weeks of the fourth trimester.^132^ Beyond postpartum surveillance, these clinics may also provide an opportunity for preconception cardiovascular risk assessment and optimization among women with a history of APOs who are planning future pregnancies. The joint ACOG/American Heart Association (AHA) presidential advisory underscores the importance of strengthening collaboration between obstetricians and cardiologists to ensure that APOs are systematically incorporated into cardiovascular risk assessment and long-term prevention strategies.^133^ After this initial phase, long-term follow-up should transition to the primary care physician, who plays a central role in sustained cardiovascular prevention.^115,132^ (*Figure 4*) Primary care ensures continuity over years, incorporating routine cardiometabolic screening (BP, glucose, lipids) and lifestyle counselling into regular check-ups. Patients with chronic hypertension, dysglycaemia, dyslipidaemia, or evidence of subclinical organ damage should be referred to cardiology or other specialists as indicated. This structured handoff represents the ideal integrated pathway to reduce fragmentation and secure durable improvements in women’s cardiovascular health (*Figure 4*).

### Remote monitoring and digital health Interventions

In the setting of HDP, HBPM integrated with telehealth platforms has consistently outperformed traditional office-based follow-up. Compared with routine care, HBPM programmes increase ascertainment of BP values, lower the rate of hospital readmissions, and allow for more rapid adjustment of antihypertensive therapy.^119,134,135^ Embedding data transfer into electronic health records and using automated alert systems further enhances clinical responsiveness, while ensuring equitable access for women in rural or underserved communities.^135,136^ Beyond BP, digital platforms targeting weight and lifestyle behaviours are increasingly leveraged in the fourth trimester. Postpartum programmes, delivered via mobile or web-based modules, have demonstrated clinically meaningful weight reduction (approximately 2.8–3.0 kg during the first postpartum year) and improvement in cardiometabolic risk profiles after GDM or HDP.^129–131,137^ Key components include individualized coaching, self-monitoring, goal setting, multimedia feedback, and gamification elements. Telemedicine follow-up plays a complementary role by reducing barriers such as childcare, transportation, and limited maternity leave. Remote monitoring models not only facilitate short-term stabilization in the postpartum period but also provide a sustainable framework for integrating lifestyle counselling and cardiometabolic screening into long-term preventive care.^132,138^

### Non-optimal care: missed opportunities

One of the most pervasive barriers is the insufficient knowledge among clinicians regarding the cardiovascular implications of APOs (*Figure 5*). HDP, GDM, preterm birth and small-for-gestational-age birth are all now recognized by the ACOG, AHA and European Society of Cardiology (ESC) as risk-enhancing factors for future CVD (*Figure 5*).^72,133^ Despite substantial clinical consequences, this knowledge has yet to be fully translated into routine practice, and without systematic integration of APOs into clinical risk stratification, opportunities for early intervention are likely to be missed. Surveys consistently show that many clinicians—across primary care, internal medicine, cardiology, and even obstetrics—fail to incorporate APOs into cardiovascular risk assessment. Awareness varies markedly by specialty, with cardiologists and obstetricians more likely than general practitioners to recognize the link, but overall knowledge remains inadequate.^139,140^ Similar care gaps have been reported internationally: in Australia, fewer than half of providers correctly identified HDP as a risk factor for future CVD <10 years postpartum.^141,142^ These findings suggest that improving clinician education is necessary but not sufficient. Training programmes must ensure that providers both recognize APOs as risk enhancers and feel equipped to deliver effective counselling. At the same time, this education should be embedded within codified follow-up systems that guarantee continuity of care, clear referral pathways, and long-term monitoring.

**Figure 5:**
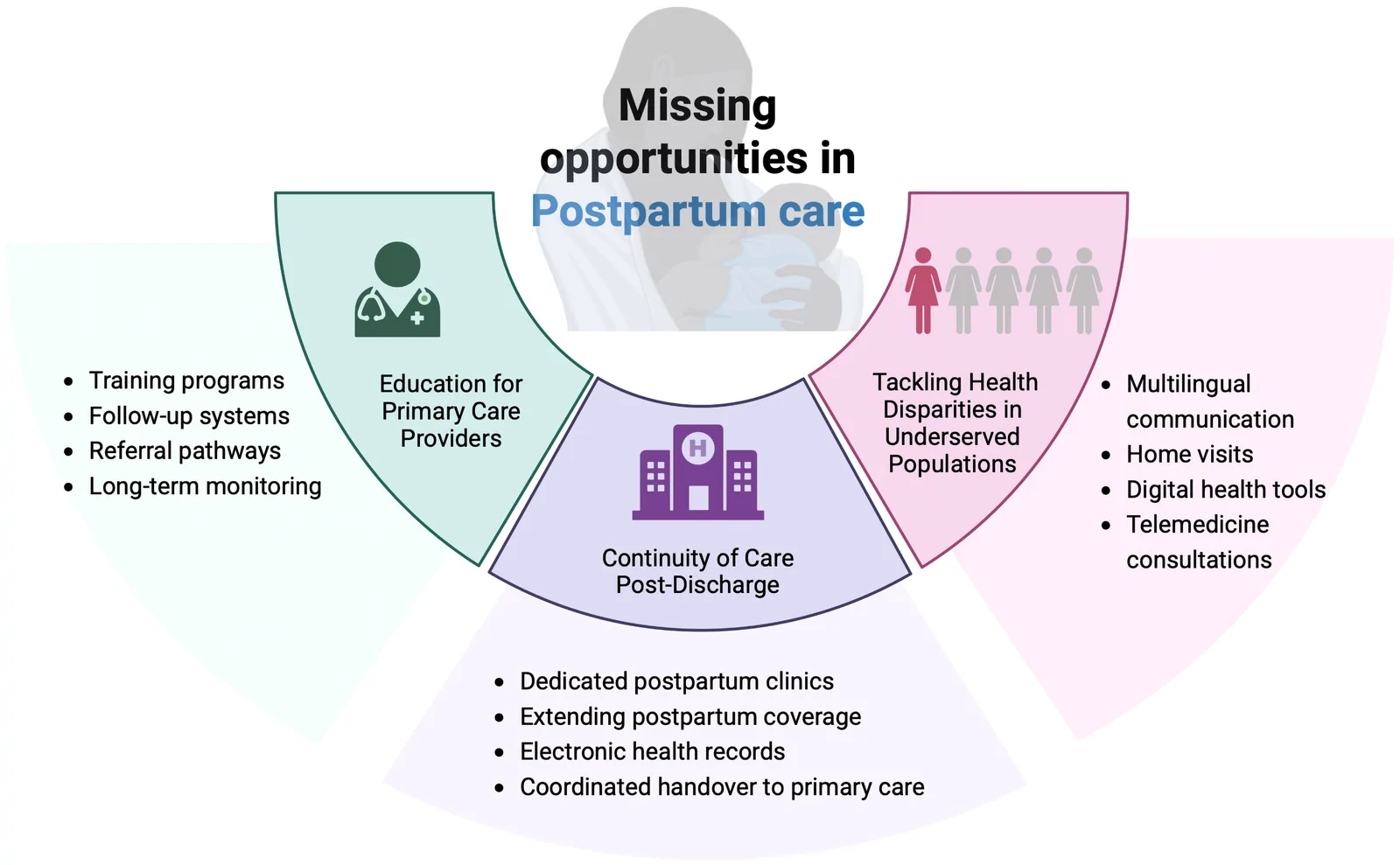
Missed opportunities for postpartum cardiovascular prevention. Conceptual framework highlighting key gaps and potential strategies to improve postpartum follow-up in women after adverse pregnancy outcomes (APOs). Three major domains are illustrated: (i) Education for primary care providers, including structured training programmes, creation of follow-up systems, clear referral pathways, and implementation of long-term monitoring; (ii) Continuity of care post-discharge, through dedicated postpartum clinics, extended postpartum coverage, use of electronic health records, and co-ordinated handover to primary care; and (iii) Tackling health disparities in underserved populations, supported by multilingual communication, home visits, and the integration of digital health and telemedicine tools to enhance access and adherence. Created in https://BioRender.com

### Continuity of care post-discharge

Continuity of care after discharge remains one of the greatest weaknesses in postpartum management (*Figure 5*). Data from large US cohorts show that up to 40% of women never attend a routine postpartum visit, and among those with APOs, only 18%–25% establish contact with a primary care provider within 6 months postpartum.^143,144^ This gap is particularly critical for women discharged on antihypertensive medication after PE: without structured follow-up, BP may remain uncontrolled and essential cardiometabolic screening—such as testing for T2D or dyslipidaemia—is often delayed or omitted, accelerating progression to chronic disease.^145^ In practice, many women re-enter the healthcare system only during a subsequent pregnancy, when complications frequently recur, or later in life when overt CVD is already present. To address this, dedicated postpartum hypertension and cardio-obstetric clinics have been developed, offering early BP monitoring, medication titration, lifestyle counselling, and co-ordinated transition to long-term care.^115^ Digital strategies add further value: randomized trials of HBPM and text-based telehealth interventions have demonstrated higher capture of BP values, fewer readmissions, improved adherence compared with standard in-office care and long-term cardiovascular enhancements.^119,135,136^ The Bridging the Chasm Collaborative is a US initiative created to close the persistent gap between obstetric and long-term women’s health, showing how structural barriers such as loss of insurance, fragmented reimbursement, and lack of coordination drive discontinuities in care.^146^ In publicly funded healthcare systems, instead of insurance coverage, reforms should focus on standardized follow-up pathways after APOs, integration of obstetric data into national health records, and mandated communication between maternity, general practice, and cardiology services. Without such structural solutions, even successful pilot programmes risk remaining isolated and failing to shift outcomes at the population level.

### Addressing health inequities after adverse pregnancy outcomes

Persistent racial, ethnic, and socioeconomic disparities remain a major challenge in postpartum cardiovascular prevention after APOs. (*Figure 5*).

APOs disproportionately affect women from some racial and ethnic groups, particularly Black women and some Asian populations. Black women experience a nearly three-fold higher maternal mortality rate than White women and have higher rates of HDP and severe maternal morbidity. Asian women carry a disproportionate burden of GDM and adverse outcomes related to GDM.^147–149^

Women from underserved racial and ethnic groups are also less likely to attend postpartum visits, less likely to receive cardiometabolic screening, and more likely to experience delays in treatment. Structural racism, socioeconomic disadvantage, such as unstable housing, food insecurity, caregiving responsibilities, limited insurance coverage, and geographic barriers, all contribute to these inequities.^150^ Remote HBPM programmes have been shown to increase BP documentation and improve postpartum visit attendance, with particular benefit among women from underserved backgrounds.^135,136^ Other solutions include telemedicine consultations, which reduce the burden of travel and childcare and have been associated with lower no-show rates and improved accessibility for women in geographically remote or resource-limited settings.^138,151^ A rapid transition to audio-based telehealth postpartum visits nearly eliminated racial disparities in hypertension follow-up rates: among Black patients, attendance increased from 48.5% pre-telehealth to 76.3% post-telehealth, reducing the racial gap to just 0.4%, whereas rates among White patients remained stable (73.1% to 76.7%).^152^ Similarly, home visits represent another strategy to bridge disparities by providing direct BP monitoring and counselling in the community; although less widely studied in postpartum hypertension, this model has been proposed as a mean of improving access and avoiding emergency readmissions.^115,153^ Because access to these programmes can be variable, digital and community-based interventions may inadvertently sustain or widen disparities unless equity is a central focus.

## Future directions for research and clinical practice

Recognizing the postpartum period as a critical preventive window for CVD in women with APOs has emerged as one of the most important conceptual shifts in maternal health.^4^ It is now well established that APOs are associated with increased risk for both short-term and long-term CVD. Pregnancy thus represents a unique ‘stress test’ for the maternal cardiovascular system, unmasking vulnerabilities that may persist long after delivery. The postpartum phase, referred to as the ‘fourth trimester,’ offers the first opportunity to prevent CVD, making it a key preventive window for the female population. Still, with APOs affecting around 20% of pregnancies, systematic cardiovascular screening and interventions for all affected women raise legitimate questions about feasibility. The most immediate opportunities for risk identification begin during pregnancy itself, when maternal physiology undergoes profound haemodynamic adaptations that can unmask latent cardiovascular vulnerability. BP trajectories with higher BP values from early gestation onwards have been consistently linked not only with the risk of developing HDP but also with the development of CHT years later.^154,155^ Because BP monitoring is already universal, inexpensive, and systematically embedded in antenatal care, it represents an ideal low-cost screening tool for future cardiovascular risk. In addition, women with HDP can exhibit echocardiographic abnormalities in the peripartum period, which may help to identify those at risk of developing persistent hypertension postpartum.^156,157^ A multivariable prediction model incorporating clinical parameters (maternal age and first-trimester BP) together with echocardiographic markers (left ventricular mass index >75 g/m^2^, relative wall thickness >0.42, and E/e′ ratio >7) showed excellent discriminatory accuracy (AUC 0.85; 95% CI: 0.79–0.90) for predicting chronic hypertension after HDP.^156^ Together, these findings highlight pregnancy as a natural and inclusive entry point into preventive cardiology, offering clinicians the ability to identify at-risk women even before birth and to plan postpartum follow-up accordingly.

The second critical checkpoint for screening arises in the postpartum period, at 3–6 months after delivery.^55,145^ By this stage, maternal physiology has largely stabilized, enabling clinicians to distinguish between transient pregnancy-related alterations and persistent cardiovascular abnormalities. Reassessment at this point provides an opportunity to evaluate whether early signals detected during pregnancy have resolved or evolved into sustained pathology. Whether APOs should be incorporated into cardiovascular risk assessment as qualitative risk-enhancing factors or as formal quantitative inputs within dedicated sex-specific risk tools remains an unresolved clinical debate. Current ACC/AHA and ESC primary prevention guidelines position APOs as risk enhancers rather than variables that independently shift a calculated risk score.^72,73^ This approach has practical appeal: it avoids the false precision of applying population-derived coefficients to a postpartum cohort for whom most validated calculators were not designed, and it preserves clinical flexibility. The competing position holds that qualitative risk enhancement is insufficiently systematic, as without a numeric score, APO history is subject to clinician discretion and is predictably under-applied in clinical practice.^48,122^ Proponents of this view argue that sex-specific risk models incorporating reproductive history would reduce the care gaps in current practice.^48,122^ Resolving this debate will require prospective validation of sex-specific models in diverse postpartum cohorts, with hard cardiovascular endpoints rather than intermediate risk markers as the primary outcome.

Once high-risk women have been identified during pregnancy and confirmed at postpartum reassessment, the next challenge is determining which preventive strategies provide the greatest long-term cardiovascular benefits. Screening is only valuable if followed by actionable interventions. The postpartum period offers multiple opportunities for targeted prevention. BP control is paramount in the fourth trimester, and remote monitoring programmes have already proven effective in reducing postpartum hypertension with long-term positive effects on maternal cardiovascular system.^119–121^ Importantly, BP monitoring should not be confined to the immediate postpartum period, but rather continued on an annual basis throughout a woman’s life after APOs, given their sustained risk of CHT and CVD. Although the strongest evidence currently supports postpartum BP monitoring after HDP, comprehensive prevention should also include management of dysglycaemia, obesity, dyslipidaemia, and other modifiable cardiometabolic risk factors across the spectrum of APOs. Lifestyle modification remains foundational,^158^ but pharmacologic agents may be necessary for women with persistent dyslipidaemias, dysglycaemia, or obesity. Statins can be considered for initiation in the postpartum period in women who meet thresholds for lipid-lowering therapy or whose risk is enhanced by APO history.^159^ However, their use remains contraindicated during lactation, despite studies showing only minimal transfer into breast milk.^160^ Aspirin, while firmly established for PE prevention during pregnancy, is not recommended for routine primary prevention; it may be considered only in selected individuals at high/very high CVD risk after bleeding-risk assessment.^73^ Observational data suggest a signal of benefit: among women with PE, prenatal low-dose aspirin was associated with a lower incidence of postpartum hypertension.^161^ By contrast, in a double-blind RCT of continuing aspirin for 6 weeks after delivery in women at risk of PE, postpartum NT-proBNP levels did not differ from placebo and rates of postpartum PE were only numerically lower, underscoring the need for larger, outcomes-powered studies.^162^ On the glycaemic/weight axis, metformin has potential postpartum utility—particularly GDM—based on the UK multicentre OMAhA feasibility randomized control trial showing that early postnatal initiation is practicable. However, this remains preliminary and metformin is not yet routine for cardiometabolic prevention in the general postpartum population.^163^ GLP-1 receptor agonists have limited postpartum and lactation data; although small series suggest minimal transfer of injectable semaglutide into human milk, most guidance advises deferring GLP-1 therapy until after lactation or using it only with individualized, specialist-led risk–benefit discussion.^164^ When used, GLP-1 receptor agonists should be started for standard indications (e.g. persistent T2D despite lifestyle ± metformin, or obesity meeting pharmacotherapy criteria) at the 3–6-month review, with reliable contraception and preconception washout planning.

Several important knowledge gaps remain. First, better risk stratification tools are needed to identify which women with APOs are most likely to develop future CVD. Second, the optimal preventive strategy remains uncertain, including the relative contribution of BP control, lifestyle modification, and pharmacological therapies. Finally, whether improvements in cardiovascular risk factors during the fourth trimester translate into sustained reductions in long-term cardiovascular events remains unknown and requires adequately powered prospective studies and randomized trials.

## Conclusions

The postpartum period should be reframed as a pivotal opportunity that links pregnancy to lifelong cardiovascular prevention. APOs affect roughly one in five pregnancies and delineate a high-risk group in which targeted screening and intervention are both feasible and warranted. Pregnancy itself yields predictive signals, such as blood-pressure trajectories and early cardiac changes, while reassessment at 3–6 months postpartum confirms persistence and refines risk stratification. Management should prioritize tight BP control with annual monitoring thereafter, anchored by lifestyle measures; pharmacotherapy can then be added where indicated, although further studies are needed to define drug-specific roles for CVD prevention after APOs. Success depends on multidisciplinary, longitudinal care which involves obstetricians, cardiologists and primary-care clinicians, with seamless transitions from antenatal services to community follow-up. This clinical approach must be matched by system-level action: integrated care pathways, reimbursement for postpartum prevention visits, postpartum coverage extension, and outcomes registries. Strategic upstream investment to prevent hypertension, dysglycaemia, and atherosclerosis after APOs is likely to reduce cardiovascular events, improving outcomes while conserving healthcare resources. Targeted interventions during the fourth trimester might help transform a period of vulnerability into one of the most powerful opportunities for lifelong cardiovascular health for women.

## Declarations

### Data Availability

No data were generated or analysed for or in support of this paper.
